# Cardiovascular drug interventions in the cardio-oncology clinic by a cardiology pharmacist: ICOP-Pharm study

**DOI:** 10.3389/fcvm.2022.972455

**Published:** 2022-09-29

**Authors:** Israa Fadhil Yaseen, Hasan Ali Farhan

**Affiliations:** ^1^Baghdad Heart Center, Medical City, Baghdad, Iraq; ^2^Scientific Council of Cardiology, Iraqi Board for Medical Specializations, Baghdad, Iraq

**Keywords:** hypertension, heart failure, risk factor (RF), treatment, breast cancer, care delivery model

## Abstract

**Background:**

Cardio-oncology is a rapidly growing field that requires a novel service design to deal with the increasing number of patients. It is reported that the volume of patients at the cardio-oncology clinic in the United Kingdom is 535 patients/5 years and in Canada is 779 patients/7 years. The pharmacist has a role in reducing the consultation time of physicians.

**Objective:**

To identify the role of a qualified cardiology pharmacist at the cardio-oncology clinic using a new paradigm based on complementary interventions with the cardiologist for the management of patients with cancer and cardiovascular risk factors and/or cardiovascular diseases (CVRF/CVD).

**Methods:**

A prospective observational study was conducted at the cardio-oncology clinic in the Medical City in Baghdad, Iraq between December 2020 and December 2021. Patients with CVRF/CVD were registered. The Iraqi Cardio-Oncology Program-Pharmacist (ICOP-Pharm) paradigm was designed to involve a qualified cardiology pharmacist for initial cardiovascular (CV) drug interventions.

**Results:**

Among 333 patients who attended our clinic over the 1-year interval, 200 (60%) CVRF/CVD cases were enrolled in the study, and of them 79 (40%) patients had CV drug interventions. A total of 196 interventions were done, including 147 (75%) cases performed by the cardiology pharmacist, and 92 (63%) of the latter were CV drug initiations. Among the total CVRF/CVD treated initially by the cardiology pharmacist, hypertension 32 (26%) and cancer therapy-related cardiac dysfunction 29 (24%) were the main types.

**Conclusion:**

The qualified cardiology pharmacist was responsible for three-quarters of the initial CV drug interventions at the cardio-oncology clinic in a complementary approach to the cardiologist. The role of the cardiology pharmacist in the ICOP-Pharm paradigm may be one of the reasons for the ability of the heart team to manage 3-fold of the patient volume when compared with those in the United Kingdom or Canada.

## Introduction

Cardio-oncology is a rapidly growing field in all aspects, including emerging training programs, clinical research and publications, and cardiac imaging (1–4). For such development, a new cardio-oncology clinic paradigm, with the involvement of pharmacists and the conduction of national and international registries, is required to optimize cardiovascular (CV) care for patients with cancer (1, 5, 6). Among the obstacles in the cardio-oncology clinic, particularly in developing countries, is the limitation of advanced cardiac imaging facilities, such as cardiac magnetic resonance. Therefore, echocardiography is mostly utilized for the evaluation of cardiac function and cardiotoxicity (7). However, performing echocardiography is time-consuming in patients with cancer due to patients’ overload and poor acoustic window related to radiotherapy and mastectomy in patients with breast cancer (3). Consequently, such factors lead to prolonged consultation time and a limited number of patients to be seen at each clinic. A patient’s medical history taken by a pharmacist is one of the solutions to reduce the time length of a physician consultation (8). In 2019, the Iraqi Cardio-Oncology Program (ICOP) was established with a mission to reduce the burden of cardiovascular disease (CVD) among patients with cancer and to emphasize the role of a qualified cardiology pharmacist within the heart team. This study aimed to identify the role of a qualified cardiology pharmacist in the management of patients with cancer and CV risk factor (CVRF) and/or CVD, including cancer therapy-related cardiac dysfunction (CTCRD) using a new cardio-oncology service paradigm based on a “complementary” or “sequential” approach with the cardiologist/cardiology fellow in training (FIT).

## Materials and methods

### Ethical approval

The study was approved by the National Research Ethics Committee.

### Study setting and population

The Iraqi Cardio-Oncology Program-Pharmacist (ICOP-Pharm) is a prospective observational study, conducted at the cardio-oncology clinic in the Medical City in Baghdad, Iraq between December 2020 and December 2021. The clinic works 1 day a week, excluding national holidays and vacations, and is staffed by a senior consultant cardiologist who is qualified in cardio-oncology, a qualified cardiology clinical pharmacist, and a cardiology FIT. The pharmacist in this study is a board-certified cardiology pharmacist, has 8 years of experience in the field of clinical cardiology and 6 years of experience as a clinical researcher and data collection officer in six international registries of the EurObservational Research Program sponsored by the European Society of Cardiology in addition to the national registries. The number of patients per clinic is 17–22 patients. The study followed the ICOP-Pharm paradigm, which was designed in this study for the management of patients with cancer and one or more CVRF/CVD including CTRCD. Based on this model, and after completing a face-to-face patient interview, the board-certified cardiology pharmacist suggested the CV drug interventions to patients with CVRF/CVD who were on suboptimal medical therapy or those who developed CTRCD. The suggested interventions are based on the recommendations of the updated American and European guidelines in the management of CVD. Then, a discussion takes place with the senior consultant cardiologist/FIT regarding the suggested interventions. Confirmation or further modification of the drug interventions will be required from the cardiologist/FIT in this setting. Therefore, a drug intervention is performed by using a “complementary” or “sequential” approach, which means that CV drug interventions are completed in three stages: (1) the cardiology pharmacist suggests CV drug interventions based on the updated international guidelines for the management of CVD, (2) the cardiology pharmacist discusses the suggestions with the cardiologist/FIT, who will agree or disagree with these suggestions, and (3) the cardiologists/FIT will check if there are any additional CV drug interventions which may be missed by the cardiology pharmacist. Finally, the patient-centered decision is taken collaboratively and the patient shares in the final decision to decide which drug to be initiated. The details of interventions are recorded by the cardiology pharmacist in the registry, including information about the scientific name of the drug, dose, and frequency. The time between each patient’s interview is gained by the cardiologist/FIT to perform echocardiography for another patient.

### Iraqi Cardio-Oncology Program-Pharmacist paradigm

**FIGURE 1:**
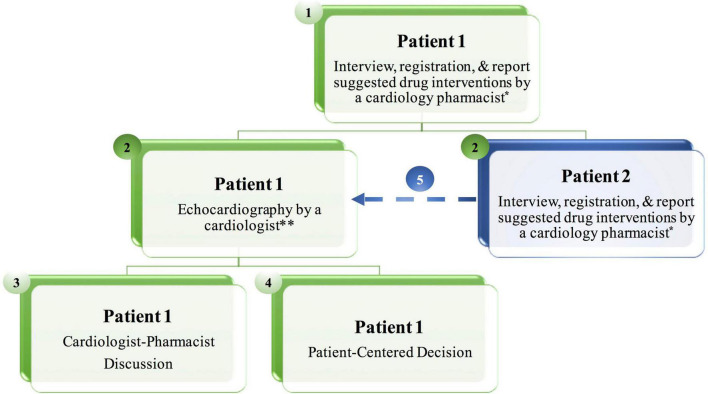
The Iraqi Cardio-Oncology Program-Pharmacist (ICOP-Pharm) paradigm at the cardio-oncology clinic. The workflow consists of five steps. During Step 2 the cardiologist performs echocardiography on the first patient, at the same time, the cardiology pharmacist meets the second patient for the same action in Step 1, resulting in less patient waiting time. *If a patient presents only for baseline echocardiography and has no cardiovascular risk factors or disease, the cardiology pharmacist performs a face-to-face interview and registration without drug interventions. ^**^When the cardiologist completes echocardiography, he informs the cardiology pharmacist about the cardiac condition, so the cardiology pharmacist adds any additional suggestions for drug interventions while the cardiologist is printing the echocardiography report before moving to Step 3.

The flow work based on the ICOP-Pharm paradigm consists of five steps. **Step 1:** The cardiology pharmacist performs a face-to-face interview with the first patient including registration, reviews home medications, and documents suggestions for cardiovascular drug intervention. **Step 2 (time-gain step):** The cardiologist performs echocardiography for the first patient. At the same time, the cardiology pharmacist meets the second patient for the same action that was mentioned in step 1. **Step 3:** The cardiology pharmacist discusses with the cardiologist patient’s medical history and the suggested drug interventions. After that, the cardiologist may add additional drug interventions that might have been missed by the cardiology pharmacist. **Step 4:** Patient-centered discussion with the first patient including patient’s opinion to initiate CV drug, selection of specific CV therapy, and patient education by the cardiology pharmacist to improve drug adherence. **Step 5:** The second patient will be ready for echocardiography and will follow other steps. During this time, the cardiology pharmacist meets the next patient (Figure 1).

### Statistical analysis

Data were represented as percentages for categorical variables and as mean and standard deviation (SD) for continuous variables, using Excel for Mac, Version 15.13.3.

## Results

**FIGURE 2:**
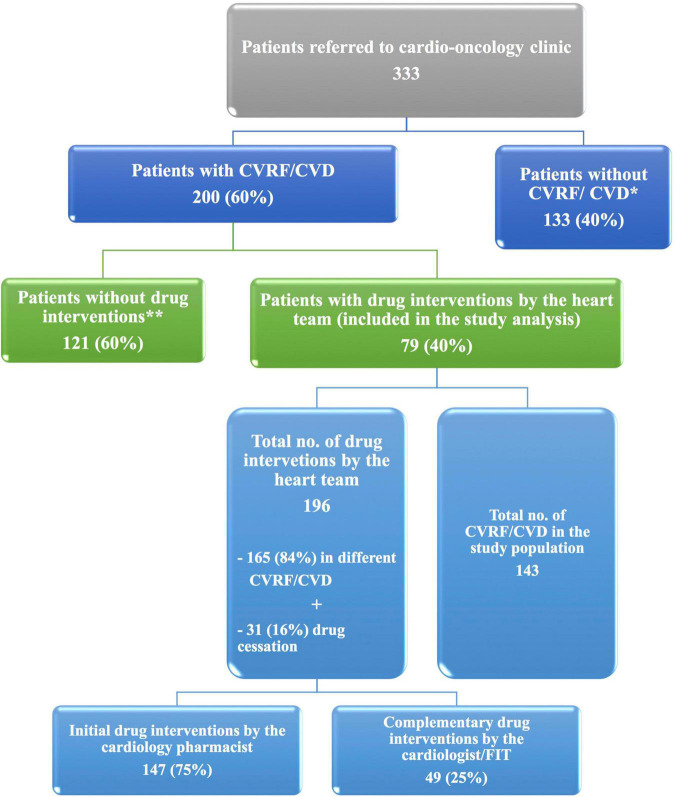
The ICOP-Pharm study flow diagram. At the cardio-oncology clinic, 200 of 333 patients with cancer had CVD/CVRF. Among patients with CVRF/CVD, drug interventions by the heart team were done for 79 patients who were included in the ICOP-Study analysis. *Patients without CVRF/CVD were referred to the clinic either for baseline echocardiography before starting cardiotoxic cancer therapy or for suspected cardiac disease. **Patients without drug interventions because they did not bring their home cardiovascular drugs and they did not know their medication name. Therefore, drug interventions were not applicable. For those patients, appointments were scheduled for drug optimization. CVD, cardiovascular disease; CVRF, cardiovascular risk factor; and FIT, fellow in training.

Among 333 patients referred to the cardio-oncology clinic over 1 year, 200 (60%) patients had CVRF/CVD, and of them, 79 (40%) patients were included in the study analysis (Figure 2).

### Characteristics of the studied population with drug intervention

Most of the patients were women 60 (76%) and among the cancer cases, breast cancer represented 46 (58%) cases, followed by gynecology cancer 10 (13%). Impaired left ventricular ejection fraction (LVEF) at baseline was found in 18 (23%) patients. More than two drug interventions were performed for 46 (58%) patients (Table 1).

**TABLE 1:** Characteristics of patients with drug interventions at the first established cardio-oncology clinic in Iraq.

Characteristics		
Age (year)	57 ± 11	
Female	60 (76)	
Cancer types	Breast	46 (58)
	Gynecology	10 (13)
	GIT	9 (11)
	Lung	8 (10)
	Other	6 (8)
Baseline LVEF (%)	Mean baseline LVEF	59 ± 14
	LVEF ≤ 40%	13 (17)
	LVEF 41–49%	5 (6)
	LVEF ≥ 50%	61 (77)
No. of CV drug interventions/patient	1	33 (42)
	2 – 4	34 (43)
	5 – 11	12 (15)
Frequency of visits to the clinic	1 visit	68 (86)
	≥2 visits	11 (14)

Values are represented as a number (%) or mean ± standard deviation (SD).

All the variables are baseline characteristics except the frequency of visits to the clinic which is estimated during follow-up.

CV, cardiovascular; GIT, gastro-intestinal tract; LVEF, left ventricular ejection fraction.

### Cardiovascular risk factors and cardiovascular diseases at baseline and post-initiation of cancer therapy

**FIGURE 3:**
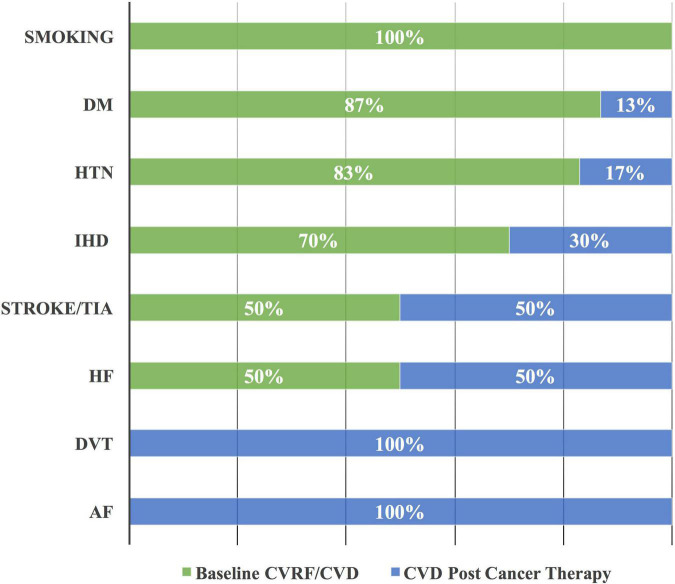
Cardiovascular risk factor/cardiovascular disease at baseline and post-initiation of cancer therapy. The majority of DM, HTN, and IHD are documented at the baseline as CVRF and CVD among patients with cancer. AF, atrial fibrillation; CVD, cardiovascular disease; CVRF, cardiovascular risk factor; DM, diabetes mellitus; DVT, deep vein thrombosis; HF, heart failure; HTN, hypertension; IHD, ischemic heart disease; and TIA, transient ischemic attack.

A total of 143 CVRF/CVD cases were enrolled, including 60 (42%) cases with hypertension (HTN), 23 cases (16%) with diabetes mellitus, and 20 cases (14%) with ischemic heart disease (IHD) (Table 2). Smoking status was reported at baseline only. A majority of 20 (87%), 50 (83%), and 14 (70%) cases, of those with diabetes mellitus, HTN, and IHD, respectively, were diagnosed at baseline (Figure 3). However, all the cases of atrial fibrillation and deep vein thrombosis were diagnosed post-initiation of cancer therapy.

**TABLE 2:** Frequency of CVRF/CVD among the study population.

CVRF/CVD	Total No. (%)
HTN	60 (42)
DM	23 (16)
IHD	20 (14)
HF	18 (13)
Smoker	10 (7)
Stroke/TIA	6 (4)
AF	4 (3)
DVT	2 (1)
Total	143 (100)

Hypertension and DM were the main CVRF, and IHD and HF were the most CVD among the study population.

AF, atrial fibrillation; CVD, cardiovascular disease; CVRF, cardiovascular risk factor; DM, diabetes mellitus; DVT, deep vein thrombosis; HF, heart failure; HTN, hypertension; IHD, ischemic heart disease; TIA, transient ischemic attack.

### Types of cardiovascular drug interventions

Types of CV drug interventions were divided into four main categories with their sub-types (Table 3 and Supplementary Table 1). The main categories were: dose titration, switch between CV drugs, cessation of one or more CV drugs, and CV drug initiation for optimizing CV drug therapy. Most types of interventions were drug initiation 135 (69%), followed by drug cessation 31 (16%) (Table 4).

**TABLE 3:** Types of drug interventions.

Main type	Sub-type
1. Titration	A. Up
	B. Down
2. Switch	A. Guideline
	B. Co-morbid
3. Cessation	A. Adverse effect
	B. No indication
	C. Drug-drug interaction (Drug class interaction)
4. Initiation	A. Initiation
	B. Re-initiation
	C. Add-on
	D. Add PPI to antiplatelet
	E. Diuretic to treat NSAID volume overload or cardiotoxicity volume overload

The main types and sub-types of drug interventions are classified after study data analysis. NSAID, non-steroidal anti-inflammatory drug; PPI, proton pump inhibitor.

**TABLE 4:** Frequency of the main types of drug interventions by the heart team.

Type of drug intervention	Total no. (%)
Initiation	135 (69)
Cessation	31 (16)
Titration	19 (10)
Switch	11 (6)
Total	196 (100)

Drug initiation is the main type of cardiovascular drug intervention by the heart team members at the cardio-oncology clinic.

### Complementary drug interventions

**FIGURE 4:**
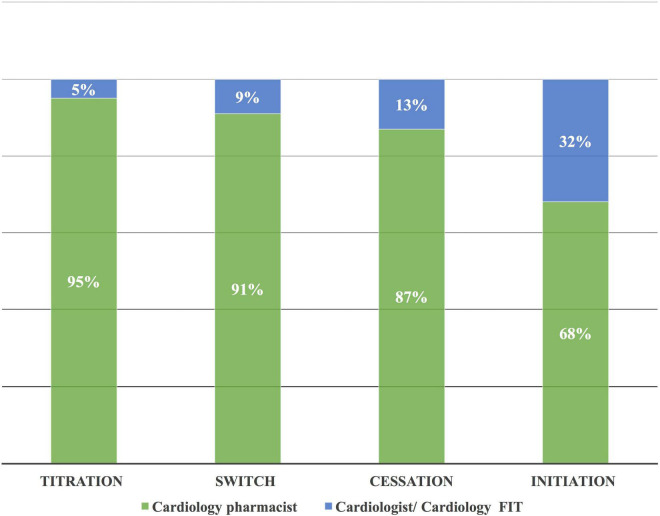
Complementary drug interventions according to the main types of interventions. The cardiology pharmacist performed most of the drug interventions due to her initial interventional complementary role in the heart team with the cardiologist/FIT. FIT, fellow in training.

**FIGURE 5:**
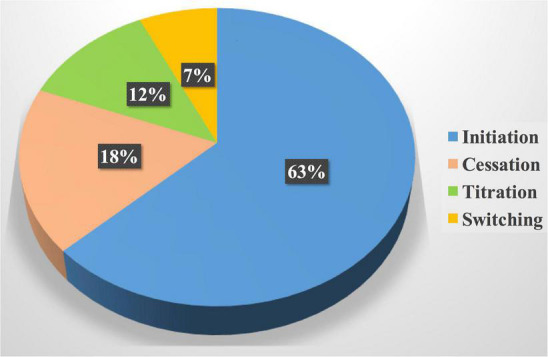
Drug interventions by the cardiology pharmacist. Drug initiation was the most common drug intervention by the cardiology pharmacist; it represented more than one-half of the total interventions followed by cessation of unnecessary cardiovascular drugs or drugs that exacerbate the cardiovascular disease.

**FIGURE 6:**
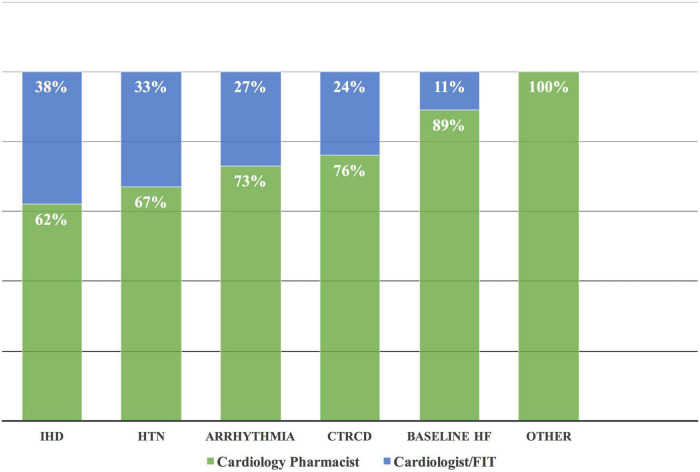
Complementary drug interventions in different CVRF/CVD. Based on the complementary approach, most of the drug intervention by the cardiology pharmacist was in patients with baseline HF followed by CTRCD. Others included transient ischemic attack, hypertriglyceridemia, and right atrial thrombus. CVD, cardiovascular disease; CTRCD, cancer therapy-related cardiac dysfunction; CVRF, cardiovascular risk factor; HF, heart failure; HTN, hypertension; IHD, ischemic heart disease.

**FIGURE 7:**
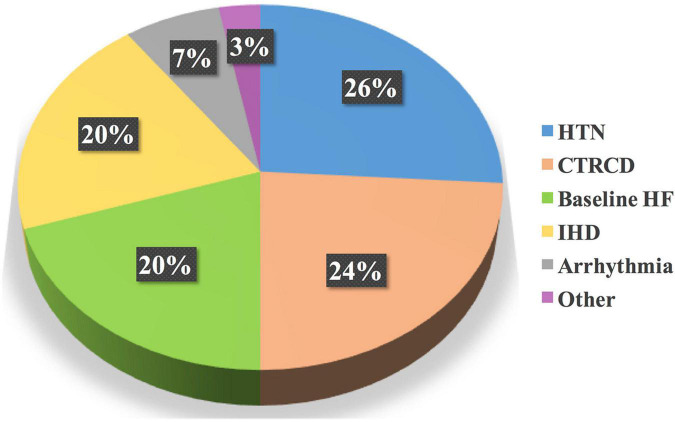
Most common CVRF/CVD treated initially by the cardiology pharmacist. Based on the total drug interventions by the cardiology pharmacist, HTN, followed by CTRCD, were the main CVRF/CVD cases treated initially by the cardiology pharmacist. CVD, cardiovascular disease; CTRCD, cancer therapy-related cardiac dysfunction; CVRF, cardiovascular risk factor; HF, heart failure; HTN, hypertension; IHD, ischemic heart disease.

The heart team performed 196 drug interventions on 79 patients at the cardio-oncology clinic (Figures 2, 4). Among the 147 (75%) drug interventions performed by the cardiology pharmacist, drug initiation was the most common type of intervention reaching 92 (63%), while switching between CV drugs was the least performed type with only10 (7%) interventions (Figure 5). Of 165 CVRF/CVD reported cases in the complementary interventions by the heart team, 120 (73%) cases were optimized initially by the cardiology pharmacist, mainly in 31 HTN (26%) and 29 CTRCD (24%) (Figures 6, 7).

### Drug interventions based on cardiovascular risk factors/cardiovascular diseases

**FIGURE 8:**
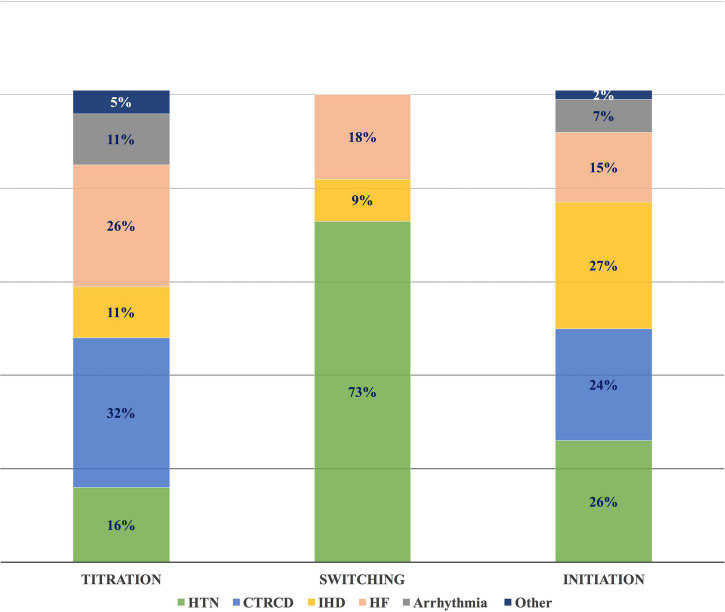
Frequency of different CVRF/CVD in different types of drug interventions. The most common drug interventions in different CVRF/CVD cases were dose titration in CTRCD, switching between drugs in HTN, and drug initiation in IHD. CVD, cardiovascular disease; CTRCD, cancer therapy-related cardiac dysfunction; CVRF, cardiovascular risk factor; HF, heart failure; HTN, hypertension; IHD, ischemic heart disease.

The most common CVRF/CVD-associated cases with drug interventions were 46 HTN (28%), 39 IHD (24%), and 38 CTRCD (23%) (Table 5). CV drug initiation was mainly for IHD, HTN, and CTRCD, with a frequency of 36 (27%), 35 (26%), and 32 (24%), respectively. Among 135 drug initiations, angiotensin-converting enzyme inhibitors (ACEI) and beta-blockers were the most common drugs used, with 37 (27%) and 39 (25%), respectively. Switching between CV drugs was widely done in patients with HTN; 8 (73%). While dosing titration was substantially performed in 6 CTRCD cases (32%), and 5 heart failure (HF) cases (26%) (Figure 8).

**TABLE 5:** Frequency of drug interventions by the heart team in different CVRF/CVD.

CVRF/CVD	Total no. of drug interventions (%)
HTN	46 (28)
IHD	39 (24)
CTRCD	38 (23)
Baseline HF	27 (16)
Arrhythmia	11 (7)
Other	4 (2)
Total	165 (100)

CV drug interventions are performed mainly in HTN followed by IHD and CTRCD.

CVD, cardiovascular disease; CTRCD, cancer therapy-related cardiac dysfunction; CVRF, cardiovascular risk factor; HF, heart failure; HTN, hypertension; IHD, ischemic heart disease.

### Cessation of drugs

**FIGURE 9:**
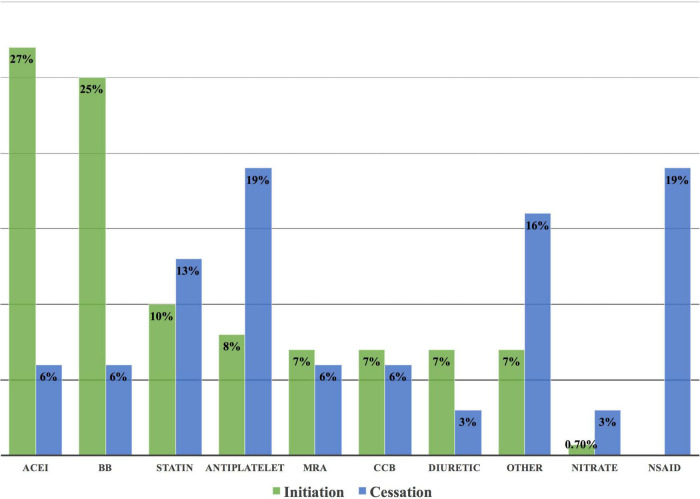
Frequency of drug cessation and initiation at the cardio-oncology clinic. Non-steroidal anti-inflammatory drugs and antiplatelets were the most common drugs to be stopped at the cardio-oncology clinic due to the lack of indication for their use or due to exacerbation of the underlying cardiovascular disease or risk factor. ACEI and beta-blockers were the most common drugs to be initiated at the clinic because of their integral role in the management of CVRF/CVD, in addition to cancer therapy-related cardiac dysfunction. ACEI, angiotensin-converting enzyme inhibitors; BB, beta-blockers; CCB, calcium channel blockers; NSAID, non-steroidal anti-inflammatory drug; MRA, mineralocorticoid receptor antagonist.

Among 31 drug cessations, non-steroidal anti-inflammatory drugs (NSAIDs) and antiplatelets were the most common medicines to be stopped, each in 6 (19%) cases (Figure 9).

### Examples of drug interventions from the real-world practice

Examples for each type of drug intervention were reported from real-world practice during drug optimization at the cardio-oncology clinic (Supplementary Table 1).

## Discussion and conclusions

The Iraqi Cardio-Oncology Program-Pharmacist is a 1-year experience study focused on the CV drug interventions by the heart team for patients with cancer and CVRF/CVD. It showed the important role of the cardiology pharmacist in the management of cardiac conditions, including the newly diagnosed CTRCD.

### Available evidence about the role of a qualified cardiology pharmacist in cardio-oncology

To date, there is no published data about the role of a qualified cardiology pharmacist in the management of patients at the cardio-oncology clinic. Usually, cardiologists are responsible for CV drug optimizations at such specialized clinics (9, 10).

### Patients overload at the cardio-oncology clinic

The volume of patients at our cardio-oncology clinic in this study was 333 patients/1 year; about 3-fold compared with those in the cardio-oncology clinic at the Royal Brompton Hospital, London, United Kingdom and the clinic at the Ottawa Hospital, Canada (9, 10). The number of patients in the clinic at the Royal Brompton Hospital over 5 years was 535 patients (average 107 patients/year), and in the clinic at the Ottawa Hospital, it was 779 over 7 years (average 111 patients/year) (9, 10). The explanations for the ability to deal with the 3-fold patient volume might be interpreted by two factors. First, different service models at the clinics; in the United Kingdom, a one-stop day case service, and in Canada, one afternoon weekly, while in Iraq one day per week. Second, the impact of initial CV drug interventions by the qualified cardiology pharmacist in a sequential approach with the cardiologist saves time for the management of a larger number of patients with cancer.

### Gender, cancer diagnosis, cardiovascular risk factors, and cardiovascular diseases

More than two-thirds of the patients were women, in line with the results in both the United Kingdom and Canada, where female patients accounted for 55.8 and 66% of the cases, respectively (9, 10). This finding is closely related to breast cancer which is the leading type of malignancy presented to our clinic as they constituted more than half of the patients in this study. Similarly, they represented 52% of the Canadian group, whereas, in the United Kingdom, they represented 30% of the cases (9, 10). Female gender is a known risk factor for anthracyclines-related cardiotoxicity, which might be explained by the lower hepatic clearance of anthracycline compared with men (11, 12). In our study, HTN was the main baseline CVRF, and similarly, UK data showed that HTN was the main CVRF among patients with cardio-oncology (33.8%), while in Canada it ranked second (43%) after smoking (45%) (9, 10). Responses to a survey conducted in Iraq revealed that HTN was reported by 71% of oncologists as the most common baseline CVRF in patients with cancer (13). Therefore, baseline CV risk assessment using HFA/IC-OS proformas, which includes HTN in all its six proformas, and optimal management are paramount for better patient outcomes (14, 15). In general, the rate of blood pressure control in patients with HTN ranges between 30 and 45% (16, 17). However, involving a pharmacist in the heart team could increase patients’ adherence to antihypertensive agents and improve blood pressure control (16, 18). Interestingly, it was shown that telemonitoring with a pharmacist had increased the blood pressure control at 6-month follow-up to 57.2% and at 12 months to 71.2% (16).

### Cardiovascular drug interventions with a complementary approach

Based on the complementary approach, it was found that the cardiology pharmacist was responsible for three-quarters of total CV drug interventions. Moreover, in reference to the type of CVRF/CVD, the cardiology pharmacist optimized more than three-quarters of baseline HF, CTRCD, and arrhythmia cases. The predominant role of the cardiology pharmacist is explained by the initial sequential approach that is used in the current paradigm for the optimization of CV drug therapy among patients with cancer. While at the cardio-oncology clinic in the United Kingdom or Canada, the cardiologists are responsible for the CV drug interventions (9, 10). Among total CV drug interventions completed by the cardiology pharmacist, initiation was the highest type of intervention, followed by cessation, titration, and then switching.

### Cardiovascular drug initiation

Cardiovascular drug initiation was the most common intervention by the heart team and by the cardiology pharmacist in all types of CVRF/CVD. It was performed in more than two-thirds of the patients at the cardio-oncology clinic mainly in patients with IHD, HTN, and CTRCD. Of note, CV drug interventions were done with a similar rate (41%) in Canada (10). Regarding CTRCD, CV drug intervention was similar to that of the United Kingdom; 23.2%, among all of the study population (9). In agreement with the report from Canada, the most common CV drugs initiated in the cardio-oncology clinic were ACEI and beta-blockers, as ACEI and beta-blockers were used in 18 and 12% of cases, respectively, and were used in combination in 14% of patients (10). Notably, ACEI and beta-blockers have an integral role as cardioprotective agents in CTRCD, and in the management of HF, IHD, and HTN (14, 19–22).

### Cardiovascular drug dose titration

Dose titration was the second intervention in all cases of CVRF/CVD except in HTN. Dose titration was done mainly for patients with CTRCD followed by HF. Early management of CTRCD with CV drug optimization during follow-up is associated with LVEF recovery (23, 24). Clinical pharmacists have an essential role at the HF clinic in the initiation and titration of guideline-directed medical therapy collaboratively with the HF team, decreasing the risk of re-hospitalization and reducing all-cause mortality by 13% (25–27).

### Cardiovascular drug switching

Switching between CV drugs is primarily performed in HTN and HF, which indicates the presence of a gap in following the guideline recommendations for managing HTN. A study in Sudan (published in 2021) at the HF clinic showed a significant role for the clinical pharmacist in the optimization of guideline-directed medical therapy, such as drug initiation, up-titration, cessation, and switching with improvement in LVEF and NYHA classes (28).

### Cessation of drugs

The pharmacist has already an integral role in reducing medication errors and preventing adverse drug events (26, 27). We found that cessation of non-indicated CV medicines or drugs that exacerbate CVD, in this study, was the second most common drug intervention by the heart team at our cardio-oncology clinic. The highest frequency for drug cessation was found with NSAIDs and antiplatelets. It is well known that NSAIDs are associated with the exacerbation of HTN and HF along with the increased risk of CVD and CV-related death (29, 30). In addition, data about the role of aspirin and other NSAIDs in reducing or increasing the risk of cancer are still controversial (29).

### Iraqi Cardio-Oncology Program-Pharmacist paradigm

The ICOP-Pharm paradigm was designed with the enrollment of a qualified cardiology pharmacist in the heart team to manage CVRF/CVD and CTRCD in patients with cancer for drug therapy optimization, reducing drug errors, and patient education to improve drug adherence. Additionally, due to a lack of regional data in this field, ICOP-Pharm enhances data registration for clinical research to improve the standards of care and ultimately the outcome. Furthermore, to collaborate in the final decision for treating patients with reference to a patient-centered approach. Thus, shortening the consultation time required by the cardiologist as well as reducing the patient’s waiting list for echocardiography, and saving time for managing a larger volume of patients. Accordingly, ICOP-Pharm proves the important role of the involvement of a qualified cardiology pharmacist at the cardio-oncology clinic, which may to some extent explain the ability to manage the 3-fold patient volume compared with that recorded in other countries.

The study is limited by the small sample size as the data were collected one time per week at our cardio-oncology clinic, which is currently the only clinic in Iraq during its first year of establishment.

In conclusion, the qualified cardiology pharmacist accomplished three-quarters of CV drug interventions at the cardio-oncology clinic in a complementary approach to the cardiologist. Most of the drug intervention by the cardiology pharmacist was drug initiation. Among the total CVRF/CVD treated initially by the cardiology pharmacist, hypertension and cancer therapy-related cardiac dysfunction were the main types. The role of the cardiology pharmacist in the ICOP-Pharm paradigm may be one of the reasons for the ability of the heart team to manage the 3-fold patient volume compared with those records from the United Kingdom or Canada.

## Data availability statement

The original contributions presented in this study are included in the article/Supplementary material, further inquiries can be directed to the corresponding author.

## Ethics statement

The studies involving human participants were reviewed and approved by the National Research Ethics Committee. The patients/participants provided their written informed consent to participate in this study.

## Author contributions

IY and HF contributed equally in the study design, writing, and review and approved for publication. IY was responsible on data collection and performed the statistical analysis. Both authors contributed to the article and approved the submitted version.

## Abbreviations

ACEI: angiotensin-converting enzyme inhibitor
CTRCD: cancer therapy-related cardiac dysfunction
CV: cardiovascular
CVD: cardiovascular disease
CVRF: cardiovascular risk factor
FIT: fellow in training
HF: heart failure
HTN: hypertension
ICOP-Pharm: Iraqi cardio-oncology program-pharmacist
IHD: ischemic heart disease
LVEF: left ventricular ejection fraction
NSAID: non-steroidal anti-inflammatory drug
UK: United Kingdom.
