# Supplementary material for: Cardiovascular drug interventions in the cardio-oncology clinic by a cardiology pharmacist: ICOP-Pharm study
Source: Front Cardiovasc Med. 2022 Sep 29;9:972455. doi: 10.3389/fcvm.2022.972455 (PMC9556995; doi:10.3389/fcvm.2022.972455)
Supplement: Supplementary file 1 [file Table_1.DOCX]

**Supplementary Table 1: Example for Different Types and Sub-types of Drug Interventions**

| Types of drug interventions | | Example |
| --- | --- | --- |
| 1. Titration | **A. Up** | - Up-titration of beta-blockers for HF - Up-titration of antihypertensive agent e.g., ACEI - Up-titration of cardioprotective agent |
|  | **B. Down** | - Down-titration of CCB because of leg edema - Down-titration cardioprotective beta-blocker because of bradycardia - Down-titration before tapering of beta-blocker which was used as first-line anti-hypertensive agent in a patient with no compelling indication and has uncontrolled blood pressure |
| 2. Switch | **A. Guideline** | - Switch methyldopa (5^th^ line anti-hypertensive agent) to ACEI (1^st^ line) for HTN treatment in patients with no contraindications - Switch atenolol to ACEI for HTN treatment in patients with no co-morbid disease and uncontrolled blood pressure |
|  | **B. Co-morbid** | - Switch rosuvastatin to atorvastatin for a patient with CVD and breast cancer |
| 3. Stop | **A. Side effect** | - Stop beta-blockers because of symptomatic bradycardia - Stop ACEI because of symptomatic hypotension with blood pressure of 79/54 mmHg - NSAID suppository, the patient was using it for long period and thought it was a laxative, then the patient developed volume overload and acute decompensated HF. - Stop hydrocodone because the patient developed side effects (tachycardia, constipation, dry mouth), the patient used the drug mistakenly for a long time as an analgesic |
|  | **B. No indication** | - Stop statin because the patient has only a history of HTN - Stop aspirin because of no indication. The patient used it in accordance with a belief that it can prevent heart disease and stroke - Stop of diuretic because the patient has no volume overload |
|  | **C. Drug-Drug interaction** | - Stop one of two ACEIs (captopril and perindopril), two ARBs (valsartan and losartan), or two beta-blockers (atenolol and bisoprolol) used at the same time. Mistakenly, the patient had used two brands of the same drug thinking that they are different medicines, due to a common attitude of visiting more than one physician. (Jumping between the private and public clinics) |
| 4. Initiation | **A. Initiation** | - Initiate cardioprotective beta-blockers and ACEI for newly diagnosed CTRCD - Initiate aspirin for a patient with a known history of MI - Initiate SGLT2 inhibitor among other anti-failure agents for a patient diagnosed with HF with EF 40% at baseline before starting cancer therapy - Initiate rivaroxaban for AF with CHA2DS2-VASc score 5 |
|  | **B. Re-initiation** | - Re-initiate cardioprotective ACEI and beta-blockers after stopping them by a physician at a private clinic - Re-initiate anti-ischemic agents because the patient stopped all of them after 1-year from PCI procedure by misunderstanding the cardiologist’s instructions - Re-initiated ACEI for HTN during follow-up as the patient expected that HTN was cured and decided to stop the medications. - A patient with baseline HF, HTN, and MI had stopped all the medications after being diagnosed with cancer. The patient personally afraid of the interaction between the baseline medicines and anticancer therapy |
|  | **C. Add-on** | - Add ACEI to CCB to control blood pressure |
|  | **D. Add PPI** | - initiate PPI because the patient was using aspirin for established MI and developed melena - Initiate PPI with aspirin for ischemic HF. |
|  | **E. Add Diuretic** | - Initiate diuretic for leg edema induced by NSAID - Initiate diuretic to treat volume overload associated with CTRCD |
| Some of the pragmatic examples of the drug interventions performed at the cardio-oncology clinic to focus on them in the future teamwork interventions at other cardio-oncology clinics.  AF, atrial fibrillation; ACEI, angiotensin-converting enzyme inhibitor; ARB, angiotensin receptor blocker; CCB, calcium channel blocker; CVD, cardiovascular disease; HF, heart failure; HTN, hypertension; NSAID, non-steroidal anti-inflammatory drug; MI, myocardial infarction; PCI, percutaneous coronary intervention; PPI, proton pump inhibitor; SGLT2, sodium-glucose transporter-2 | | |
